# Does Giving and Receiving Helping Behavior Fit Matter? The Role of Neighboring Behavior Fit in Working Residents' Mental Health

**DOI:** 10.3389/fpubh.2022.863327

**Published:** 2022-06-24

**Authors:** Jing Xiu, Zhenduo Zhang, Youqing Fan, Junwei Zheng

**Affiliations:** ^1^School of Applied Economics, University of Chinese Academy of Social Sciences, Beijing, China; ^2^School of Economics and Management, Dalian University of Technology, Dalian, China; ^3^School of Business, Western Sydney University, Sydney, NSW, Australia; ^4^Faculty of Civil Engineering and Mechanics, Kunming University of Science and Technology, Kunming, China

**Keywords:** receiving neighboring behavior, giving neighboring behavior, work-family interference, labile self-esteem, mental health

## Abstract

Ecological systems theory suggests that for individuals, the three domains of community, family, and work are connected and transfer resources among each other. In the community, residents receive and give helping behavior from and to their neighbors. Neighboring behavior underlies interactions among residents in the community, thereby influencing the work and family domains. Building on ecological systems theory, the authors propose that the compatibility of receiving and giving helping behavior among working residents is related to their mental health. Additionally, the authors propose that this congruence effect functions through work-family interference and meaning in life. Using a two-stage field questionnaire survey, this study collected data from 220 full-time Chinese working residents. Using polynomial regression and response surface analysis, receiving-giving neighboring behavior fit was found to be positively associated with mental health. Furthermore, receiving-giving neighboring behavior fit enhances mental health by decreasing work-family interference and promoting meaning in life. When giving and receiving neighboring behavior are imbalanced, working residents have higher levels of mental health when they received more neighboring behavior than they gave, in comparison to the condition when they gave more neighboring behavior than they received. Work-family interference represents inter-role conflict in which pressures from the family and work domains are mutually incompatible. Including both work to family interference and family to work interference, work-family interferences reflect the stress that working residents experience in their family and work domains. By exploring the mediating role of work-family interference, this study shows how the spillover of the benefits of neighboring behavior into the family and work domains enhances working residents' mental health. This study highlights the importance of balancing receiving and giving neighboring behavior for maintaining mental health, thus contributing both theoretically and practically to ecological systems theory.

## Introduction

Communities are considered to be places where social capital and psychological resources are cultivated to maintain mental health (1, 2). Perkins et al. (3) put forward the concept of neighboring behavior, representing both the receiving and giving of various kinds of assistance from and to neighbors. Neighboring behavior aims to resolve and prevent both current and potential related problems, such as coping with emergencies and seeking advice to resolve personal problems. Compared with the physical community environment, Zhang et al. (4, 5) and Zu et al. (6) highlighted the benefits of informal mutual assistance and information sharing among neighbors in maintaining and enhancing residents' mental health.

With increasing work pace, employees are facing increasing stress from both the work and family domains. In particular, the disruption caused by the COVID-19 pandemic and the widespread use of information communication technologies has resulted in most employees choosing to work from home and adopting teleworking (7). This choice provides employees with autonomy in scheduling their tasks (8) but stimulates enhanced work-family interference. Hunter et al. suggest that working from home results in boundary violations between work and family, thereby leading to work-family interference (9). Organizational psychology scholars have attempted to develop strategies for resolving this dilemma via enhancing family-supportive supervision, nurturing leader compassion, and decreasing technology overload and invasion (10–12). Only a few studies have focused on the influences of neighboring behavior on resolving work-family interference and maintaining mental health (4, 6).

Although prior studies have addressed the benefits brought by receiving and giving neighboring behavior on enhancing mental health, the reciprocal nature of neighboring behavior should be further addressed (13). Social exchange theory and reciprocal norms drive residents to provide support to their neighbors after having received assistance from them. Moreover, prosocial behavior has been identified as causing negative side effects for actors (14). For example, Gabriel et al. showed that helping behavior may result in ego depletion in helpers (15). Zhang et al. suggested that the potential reason for the detrimental effects of prosocial behavior may be the neglect of the resources owned by helpers (5). In the community context, Voydanoff (16, 17) suggested that helping neighbors is a commandment for working residents. The reason for this is that helping neighbors requires working residents to devote time to communicating with their neighbors and aiding them in resolving related problems. As a result, less time is available for working residents to recover from their work fatigue. Receiving neighboring behavior is a resource for working residents, which develops social adaptability and prevents mental illness (1, 18). Exploring the influences on mental health of (in)congruencies in receiving and giving neighboring behavior provides a novel perspective to explain both the shortcomings and advantages of neighboring behavior. It can also provide a potential explanation for the formerly paradoxical relationship between giving neighboring behavior and mental health. Therefore, it is necessary to explore the joint influences of receiving and giving neighboring behavior, specifically the fit between them, on mental health.

Furthermore, the current study adopts work-family interference and meaning in life as chain mediators to explore how receiving and giving neighboring behavior fit impacts mental health. Ecological systems theory provides a theoretical framework for analysing the spillover effects of neighboring behavior on psychological states in both the work and family domains (1). Ecological systems theory suggests that community, work, and family are three basic components of the personal ecological systems, which transfer resources and energies among each other. This transformation of resources plays a key role in shaping individuals' mental health (5). Receiving and giving neighboring behavior fit represents the extent to which working residents' receiving and giving neighboring behavior match. A higher level of such a fit provides working residents with ample community resources, which can spill over into their family domains. Therefore, this study adopts work to family interference and family to work interference to uncover the underlying spillover mechanism through which receiving and giving neighboring behavior fit affects mental health. Meaning in life is essential for individuals to maintain mental health and has been used as a mediator in the relationship between work-family interference and mental health (19, 20). Based on prior studies, this study further adopts meaning in life to link work-family interference to mental health.

**Figure 1:**
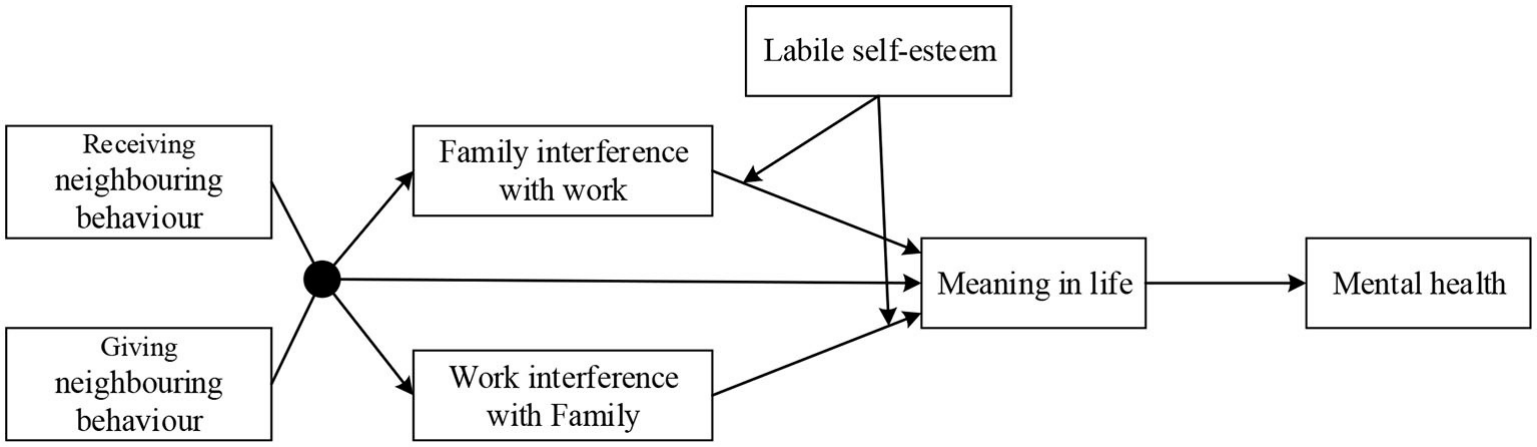
Conceptual Model.

Ecological systems theory suggests that personality traits shape the spillover process in which community resources are transferred to the work and family domains (4). Labile self-esteem is one of the basic personality traits associated with psychological syndromes such as depression and anxiety (21). In this vein, this study adopts labile self-esteem as a boundary condition to assess when receiving and giving neighboring behavior fit is beneficial for enhancing mental health. The conceptual model is depicted in Figure 1.

To test the conceptual model, the present study employed a two-wave field questionnaire survey and collected data from 220 full-time Chinese working residents. Polynomial regression with surface response analysis was employed to explore the impacts of giving and receiving neighboring behavior fit on mental health. By doing so, this study provides three contributions to both neighboring behavior theory and ecological systems theory. First, this study extends our understanding of the antecedents of mental health by examining the impacts of receiving and giving neighboring behavior fit on mental health. Second, this study uncovers the spillover process through which receiving and giving neighboring behavior fit enhances mental health by examining the serial mediating roles of work-family interference and meaning in life. Third, this study explores the boundary condition under which receiving and giving neighboring behavior fit is more or less beneficial for facilitating mental health by examining the moderating role of labile self-esteem.

## Literature Review and Hypothesis Development

### Congruence in Receiving and Giving Neighboring Behavior and Mental Health

Receiving neighboring behavior reflects the amount of assistance obtained from neighbors when confronted with difficulties (3). Prior studies have highlighted the benefits of receiving neighboring behavior on mental health. For instance, Zu et al. suggested that assistance from neighbors will relieve work-family conflicts, thereby enhancing mental health (22). Giving neighboring behavior is a process in which residents spend time helping others to resolve problems in the community (4). Recently, the giving of neighboring behavior has been highlighted as an effective tool for cultivating community resources. Zhang et al. explored the relationship between the giving of neighboring behavior and social functioning (5). Research suggests that helping neighbors rewards the helpers with cognitive information processing abilities and enhances emotional regulation skills (5), both of which are essential for maintaining mental health.

Integrating the above statements, the authors assume that when receiving and giving neighboring behavior are highly congruent, residents both receive support from neighbors and obtain resources by helping their neighbors. Under this context, residents have sufficient resources, such as social capital and positive emotions, to enable them to maintain their mental health. Because of a low level of receiving neighboring behavior, such residents are less likely to receive social and psychological resources from the community when confronted with difficulties. They have no extra access to social support and psychological resources, which leads to an increased risk of mental illness. Due to the low level of giving neighboring behavior, working residents cannot cultivate good reputations and favorable social relationships. These working residents are usually unfamiliar with their neighbors and more likely to experience mental illness due to a lack of social interactions (3). As a result, when the congruence in receiving and giving neighboring behavior is at a low level, residents are usually not involved in the community and are isolated from their neighbors, which is not beneficial for them to maintain their mental health. Hence, the following is hypothesized:

**Hypothesis 1**. *Residents have a higher level of mental health when their congruence in receiving and giving helping behavior is at a high rather than a low level*.

### Incongruence in Receiving and Giving Neighboring Behavior and Mental Health

As aforementioned, helping behavior from neighbors helps residents to release stress associated with their daily life and work. For example, Griggs et al. (1) highlighted the importance of community support for attenuating work-family conflict for low-income workers. Supporting this, Zu et al. (22) suggested that support from neighbors may help low-income residents to cope with work-family conflict, thereby enhancing their mental health and career satisfaction.

With regard to giving neighboring behavior, prior studies have highlighted both its advantageous and disadvantageous influences on psychological and behavioral responses. Giving neighboring behavior is beneficial because it provides helpers with enhanced social functioning, which is essential for maintaining mental health (5). Recently, several scholars have begun to focus on the negative aspects of helping behavior. Helping behavior entails individuals assigning some of their time to assist others in resolving and preventing difficulties (23). Time is a limited resource in any day (15). This means that helpers face a trade-off between completing their own tasks and helping others with their limited time. Thus, when residents consume time and other resources (e.g., cognitive and emotional resources) to help their neighbors, they are more likely to experience ego depletion (15), which is detrimental to their mental health (24).

In the aggregate, when receiving and giving neighboring behavior are imbalanced in either direction, residents will present different levels of mental health. In the condition of giving high levels of neighboring behavior while receiving low levels of neighboring behavior, residents both experience the advantages and disadvantages of helping their neighbors. In particular, residents suffer from the loss of time and other resources without benefitting from resource supplementation (i.e., receiving neighboring behavior). Under this condition, residents are more likely to suffer lower levels of mental health because of ego depletion. Under the condition of receiving high levels of neighboring behavior while giving low levels of neighboring behavior, residents receive support from their neighbors but extend little effort to help others, even though the cultivation of social support would aid them in maintaining their mental health. Hence, the following is hypothesized:

**Hypothesis 2**. *Residents have a higher level of mental health when they receive a higher level but give a lower level of neighboring behavior compared with those who receive a lower level but give a higher level of neighboring behavior*.

### Serial Mediating Roles of Work-Family Interference and Meaning in Life

#### Receiving and Giving Neighboring Behavior Fit and Work-Family Interference

Work-family interference represents a form of inter-role interference in which stress from work and stress from family play mutually incompatible roles (25). Work-family interference arises when one's ability cannot satisfy one's need to cope with both work and family demands (26). When working residents are distracted from their work by family-related responsibilities, they are experiencing family interference with work (27). By contrast, work interference with the family occurs when participation in the family role becomes more difficult because of participation in work (28). Community resources can be transferred to the family and work domains and are therefore important for resolving work-family interference.

Family demands include physical duties like fixing or repairing the home (task aspects), making family-related decisions (cognitive aspects) and taking care of a spouse, children, and parents (relational aspects) (29). Receiving neighboring behavior includes receiving help in an emergency and receiving advice on family-related matters, both of which are beneficial to fulfilling family roles (1). Also, such community resources can be delivered into the work domain. Receiving neighboring behavior nurtures residents' well-being, which can spill over into their work role and enhance their positive emotions, thereby broadening their behavioral and cognitive repertories available for fulfilling their work roles (6).

Regarding giving neighboring behavior, Zhang et al. (5) suggested that helping neighbors is a process in which working residents develop social functioning (i.e., cognitive information processing abilities and emotional regulation skills). Social functioning allows working residents to adapt their emotions to the current satiation to cope with emotional demands at work and in the family (5). Social functioning helps working residents to fulfill demands from the work and family domains through assertive communication and time management with family members or leaders (1). Thus, a high level of giving neighboring behavior facilitates the fulfillment of both work and family roles.

Taken together, when working residents experience a high level of receiving and giving neighboring behavior fit, they receive sufficient community resources (e.g., positive emotions, advice, and care) for delivery into the family and work domains. These resources facilitate working residents' fulfillment of their work and family role demands, thereby decreasing the likelihood they will experience work-family interference. Hence, the following is hypothesized:

**Hypothesis 3**. *Receiving and giving neighboring behavior fit is negatively associated with work-family interference (i.e., family interference with work and work interference with family)*.

#### Work-Family Interference and Meaning in Life

Meaning in life represents the sense made of, and significance felt regarding, the nature of one's being and existence (30). Moreover, Steger et al. identified coherence, purpose, and existential mattering as the three basic precursors of meaning in life (30). Based on this perspective, the psychological antecedents of meaning in life have been extended. Among the other psychological factors involved, positive emotions and psychological capital have received particular attention.

As aforementioned, work-family interference arises when family or work demands exceed one's capabilities (31). When confronted with work-family interference, working residents are more likely to experience emotional strain because of the consistent drain of psychological resources (32). Nauman et al. explored the positive association between work-family interference and emotional exhaustion (33). Hicks and King suggested that positive mood is a cue for individuals to make judgements about meaning in life (34). Thus, decreased positive mood caused by work-family interference undermines working residents' judgements about their meaning in life. Psychological capital is a further psychological factor that can impact meaning in life. Psychological capital enables individuals to evaluate life events positively and thus causes them to experience higher meaning in life (35). However, Pu et al. suggested that work-family interference consumes work residents' psychological capital, thereby decreasing meaning in life (36). Taken together, the following is hypothesized:

**Hypothesis 4**. *Work-family conflict is negatively associated with meaning in life*.

#### Meaning in Life and Mental Health

The relationship between meaning in life and mental health has attracted attention from scholars. For example, Shiah et al. examined the positive relationship between meaning in life and mental health in a non-clinical sample of Chinese participants (20). Li et al. found that happiness and meaning in life are two key indicators of mental health as they ameliorate perceived stress (37). Prior studies have suggested that meaning in life is one of the strongest motivations and provides people with hope when they are confronted with adversity (38). Psychological resources are obtained from meaning in life and facilitate coping with personal traumas and maintaining mental health. As such, the following is hypothesized:

**Hypothesis 5**. *Meaning in life is positively associated with mental health*.

Taken together, the authors assume that receiving and giving neighboring behavior provides working residents with the community resources needed to fulfill the demands imposed by work and family roles, thereby decreasing their likelihood of experiencing work-family interference. As a result, these working residents have more positive cues when making judgements about meaning in life, which is beneficial for enhancing mental health. Thus, the following is hypothesized:

**Hypothesis 6**. *Work-family interference and meaning in life serially mediate the relationship between receiving and giving neighboring behavior fit and mental health*.

### Moderating Role of Labile Self-Esteem

Zhang et al. suggested that the spillover processes through which community resources are delivered into the work and family domains are contingent upon personal traits (4). Moreover, Kinnunen et al. found that the association between work-family conflict and psychological well-being depends on personality (39). To better understand the boundary conditions under which receiving and giving neighboring behavior fit is beneficial for mental health, this study assumes labile self-esteem to be a moderator in the relationship between work-family interference and meaning in life, in light of its impacts on psychological well-being.

Labile self-esteem reflects an individual's tendency to experience fluctuations in their level of self-esteem (40). In contrast to self-esteem, people with a high level of labile self-esteem are more likely to shift the perception they hold of themselves, thereby exposing themselves to increased risks for depressive symptoms. Roberts and Monroe found that persons with high labile self-esteem exhibit a special sensitivity to stress (41). Moreover, Roberts and Kassel found that individuals with high self-esteem have a higher likelihood of experiencing life stress (42). Work-family interference is commonly regarded as a hindrance stressor, which consistently consumes working residents' job resources and results in decreased meaning in life (43, 44). Given the increased sensitivity to stress caused by a higher level of labile self-esteem, work-family interference exerts strong negative impacts on meaning in life for working residents with a high level of labile self-esteem. By contrast, working residents with a low level of labile self-esteem tend to cope with stress proactively and are less likely to be impacted by it. Thus, work-family interference is less detrimental to meaning in life for working residents with low levels of labile self-esteem. Hence, the following is hypothesized:

**Hypothesis 7**. *Labile self-esteem moderates the relationship between work-family interference (i.e., family interference with work and work interference with family) and meaning in life in such a way that the relationship between work-family interference and meaning in life is stronger in working residents with high rather than low levels of labile self-esteem*.
**Hypothesis 8**. *Labile self-esteem moderates the indirect relationship between receiving and giving neighboring behavior and mental health in such a way that the indirect relationship between receiving and giving neighboring behavior fit and mental health is stronger in working residents with high rather than low levels of labile self-esteem*.

## Methods

### Sampling and Procedures

This research focuses on the impacts of receiving and giving neighboring behavior fit on mental health through work-family interference and meaning in life. Two criteria for samples were set: having full-time jobs and having lived in the current communities for over 1 year. Samples were chosen randomly from people living in urban communities in Harbin City, China. With the assistance of trained social workers, questionnaires were sent out in hard copies. To control for common method variance, this study adopted a lagged questionnaire survey and collected data at two time points. The first time point was on 1st November 2018. Neighboring behavior (i.e., receiving and giving neighboring behavior) and work-family interference (i.e., family interference with work and work interference with family) were surveyed at this time point. The second time point occurred on 16th December 2018, and both meaning in life and mental health were surveyed at this time point.

A total of 317 surveys were collected in the first wave, and 220 surveys in the second wave. The effective response rate was 69%. The results of drop-out analysis indicated that the dropped samples exhibited an insignificant difference from the retained samples in regard to neighboring behavior, work-family interference, meaning in life, and mental health. Respondents worked in diverse industries (e.g., governments, manufacturing, and internet companies). On average, they were 41.26 (± 9.60) years old and had 21.77 (± 11.26) years of work experience. 41.4% of the respondents were male, and 78.6% were married. Regarding their education level, 19.1% of the samples had senior school education and below, 38.6% had high school education, 27.3% had a college education, and 15.9% had a bachelor's degree.

### Measures

All scales were originally developed in English and were translated into Chinese. To ensure their validity, the back-translation procedure suggested by Brislin was employed (45). A five-point Likert scale was used ranging from 1 = *strongly disagree* to 5 = *strongly agree* without special statements.

#### Neighboring Behavior

Ten items developed by Perkins et al. (3) were used to measure neighboring behavior. This scale included two dimensions: receiving neighboring behavior (five items) and giving neighboring behavior (five items). A sample item for receiving neighboring behavior is, “My neighbors help me in an emergency.” The Cronbach's α for this scale was 0.82 in the current study. A sample item for giving neighboring behavior is, “I help my neighbors in an emergency.” The Cronbach's α for this scale was 0.77 in the current study. A five-point Likert scale was used for the frequency with 1 = *almost never* and 5 = *always*.

#### Mental Health

Mental health was assessed via 12 items of the GHQ-12 validated by Gao et al. (46) in Chinese samples. A sample item is, “I am able to concentrate.” The Cronbach's α was 0.96 in the current study.

*Work-family interference*. Eight items developed by Grzywacz and Marks (47) were used to assess work-family interference. Family interference with work was measured by four items. A sample item is, “Stress at home makes me irritable at work”. The Cronbach's α for this scale was 0.96 in the current study. Work interference with family was measured by four items. A sample item is, “Stress at work makes me irritable at home.” The Cronbach's α for this scale was 0.95 in the current study.

*Meaning in life*. This study used five items in presence of meaning subscale developed by Steger et al. (30) and suggested by Crego et al. (48). A sample item is, “I understand my life's meaning.” The Cronbach's α was 0.89 in the current study.

#### Labile Self-Esteem

This study used five items developed by Dykman (49) to measure labile self-esteem. A sample item is, “Compared to most people, my self-esteem changes rapidly.” The Cronbach's α was 0.96 in the current study.

#### Control Variables

Considering the potential influences on mental health, the statistical analysis employed in this study controlled for gender, age, educational level, and marital status (50–52). Gender is a dichotomous variable for which 1 = *male* and 2 = *female*. Age is a continuous variable and respondents reported their age directly. Educational level is a categorical variable where 1 = *senior school degree*, 2 = *high school degree*, 3 = *college degree*, 4 = *bachelor's degree*, and 5 = *master's degree and above*. Marital status is a dichotomous variable where 1 = *married* and 2 = *other*.

## Results

### Results of Descriptive Statistics

The distribution of demographic information is depicted in Table 1. Furthermore, we have calculated the means, standard deviations, and correlations between the focal variables. Theses results are shown in Table 2.

**Table 1:** Results of descriptive statistics.

**Variables**	**Group**	***N***	**%**
Gender	Male	91	41.40
	Female	129	58.60
Education	Senior School	42	19.10
	High School	85	38.60
	College	50	22.70
	Bachelor	35	15.90
	Master and above	8	3.60
Marital sStatus	Married	173	78.60
	Others	47	21.40

**Table 2:** Results of correlation analysis.

	**1**	**2**	**3**	**4**	**5**	**6**	**7**	**8**	**9**	**10**	**11**
1. Gender											
2. Age	0.02										
3. Marital Status	0.08	−0.30^**^									
4. Education	−0.07	−0.28^**^	0.12								
5. Giving Neighboring Behavior	0.04	−0.03	0.16^*^	0.14^*^	(0.77)						
6. Receiving Neighboring Behavior	0.03	−0.05	0.15^*^	0.22^**^	0.71^**^	(0.82)					
7. Family Interference with Work	0.06	0.18^**^	−0.07	−0.46^**^	−0.31^**^	−0.35^**^	(0.96)				
8. Work Interference with Family	0.02	0.09	−0.04	−0.18^**^	−0.41^**^	−0.41^**^	0.33^**^	(0.95)			
9. Meaning in Life	−0.09	−0.09	0.08	0.27^**^	0.37^**^	0.47^**^	−0.37^**^	−0.34^**^	(0.89)		
10. Mental Health	−0.06	−0.07	0.06	0.37^**^	0.43^**^	0.55^**^	−0.41^**^	−0.41^**^	0.67^**^	(0.96)	
11. Labile Self-Esteem	0.08	0.08	−0.03	−0.17^*^	0.07	−0.02	0.05	0.07	−0.58^**^	−0.57^**^	(0.96)
Mean		41.26			1.87	1.87	3.45	2.52	3.24	3.15	2.96
SD		9.60			0.58	0.66	1.09	0.95	0.82	0.82	1.00

*N = 220;*

** *p < 0.01,*

* *p < 0.05; Values in the parentheses are Cronbach's Alpha*.

### Analysis Strategy

Polynomial regression with response surface analysis was adopted to test the hypotheses (53). This statistical method has been employed by researchers in the fields of psychology and management to explore how the combination of two independent variables is related to one dependent variable, particularly in the case of congruence and discrepancy measures (54). Dawson suggested that this approach is superior to traditional regression analysis because polynomial regression analysis can provide a three-dimensional view of the interactive influence of two predictors on one dependent variable (55).

The classical polynomial regression equation is Z = b_0_ + b_1_X + b_2_Y + b_3_X^2^ + b_4_XY + b_5_Y^2^ + e. In this equation, Z is the dependent variable (mental health), X represents giving neighboring behavior, and Y represents receiving neighboring behavior. In the response surface analysis, coefficients in the polynomial regression are used to examine the response surface pattern, which is depicted to provide a three-dimensional visual representation of the data for the interpretation of the polynomial regression results (56). The surface pattern was determined by the slope and curvature of the congruence line (X = Y) and the incongruence line (X = -Y). For Hypotheses 3 through 8, to assess the moderated mediation effect on the relationship between receiving and giving neighboring behavior fit and mental health, Edwards and Cable's (57) approach was followed first. A block variable that combined the five polynomial terms (X, Y, XY, X2, and Y2) was calculated based on their respective weights in the polynomial regression analysis (58). Then, path analysis was conducted to examine the moderated mediation model by using Mplus 7.4.

Following Edwards's (57) suggestions, X and Y were centered. To test Hypothesis 1, it was determined whether the slope along the congruence line (X = Y) was significantly positive. This would indicate that mental health increased as giving and receiving neighboring behavior matched at higher levels rather than lower levels. Regarding Hypothesis 2, it was determined whether the slope along the incongruence line (X = –Y) was significantly negative. Such a result would indicate that the working residents' mental health was better under the condition of lower giving neighboring behavior with higher receiving neighboring behavior rather than higher giving but lower receiving neighboring behavior.

### Hypothesis Testing

Polynomial regression analysis was used to determine how congruence and incongruence in receiving and giving neighboring behavior affect mental health. This analysis was conducted in Mplus 7.4. The results of Model 2 (Table 3) indicate a negative association between giving neighboring behavior and mental health (β = −0.40, *p* < 0.01). However, the association between receiving neighboring behavior and mental health was significantly positive (β = 0.96, *p* < 0.01).

**Table 3:** Results of polynomial regression with response surface analysis.

	**Mental health**			
	**Model 1**		**Model 2**	
	**B**	**SE**	**B**	**SE**
Constant	2.17	0.36	1.69	0.33
Gender	−0.07	0.11	−0.10	0.09
Age	0.08	0.12	0.04	0.10
Marital education	0.07	0.13	−0.04	0.12
Education	0.63^**^	0.11	0.41^**^	0.10
Giving neighboring behavior (X)			−0.40^**^	0.21
Receiving neighboring behavior (Y)			0.96^**^	0.18
*X* ^2^			−0.29	0.61
XY			0.01	0.94
Y^2^			0.16	0.42
*F*	8.86^**^		15.53^**^	
*R* ^2^	0.14		0.40	
Δ*R*^2^			0.26^**^	
Slope along x = y			0.56^**^	0.08
Curvature on x = y			−0.13	0.15
Slope along x = -y			−1.35^**^	0.38
Curvature on x = -y			−0.15	1.19

*N = 220;*

** *p < 0.01*.

**Figure 2:**
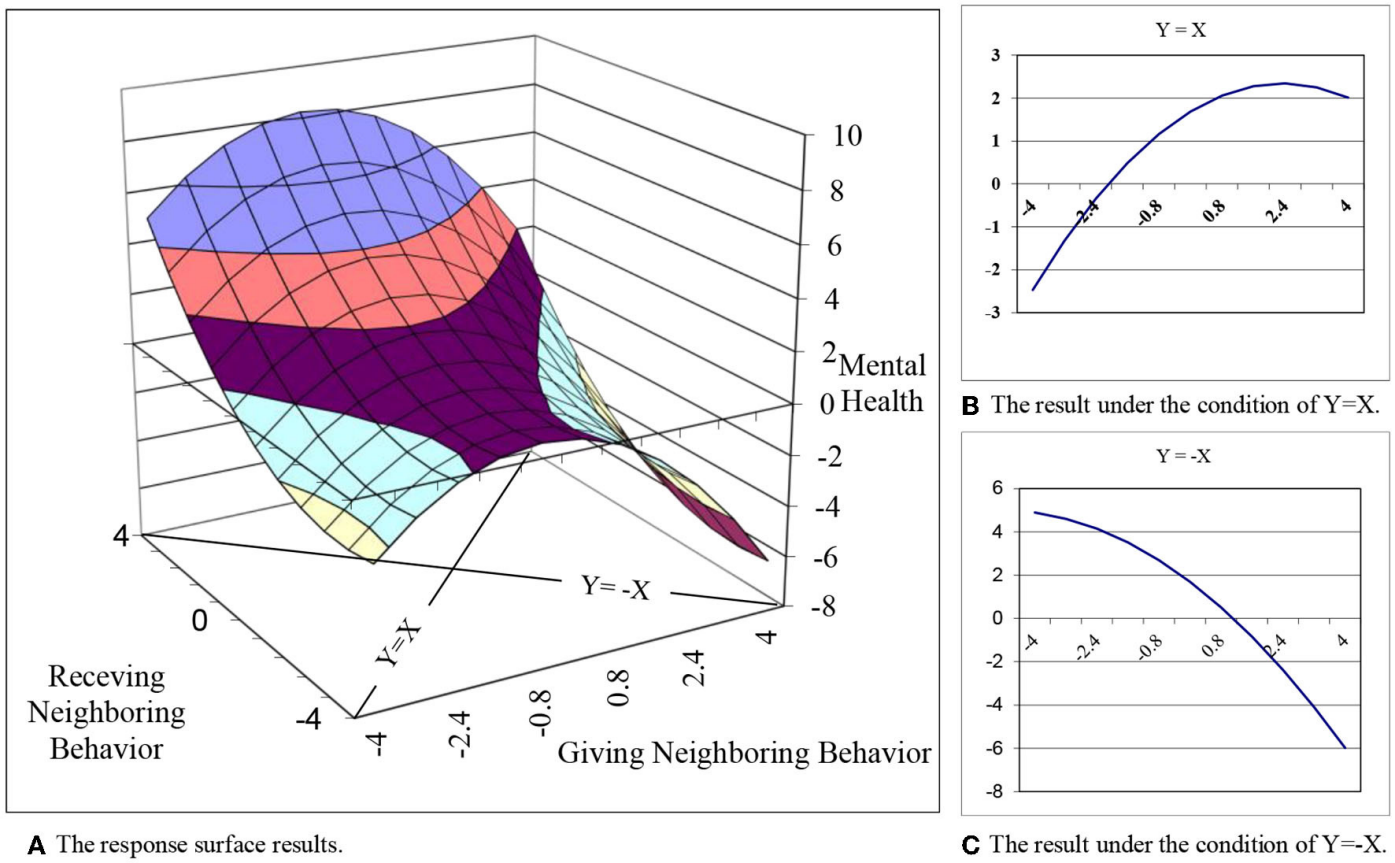
The overall response surface results.

To test both H1 and H2, response surface analysis was used and the response surface pattern was examined based on the curvature and slopes of the congruence and incongruence lines. The results shown in Table 3 indicate that the slopes of the congruence line (x = y) (β = 0.56, *SE* = 0.08, *p* < 0.01) and the incongruence line (x = –y) (β = −1.35, *SE* = 0.38, *p* < 0.01) are significant. The positive slope of the congruence line indicates that mental health was higher when giving and receiving helping behavior were congruent at higher levels. This supports Hypothesis 1. The negative slope of the incongruence line indicates that mental health was higher in the condition when working residents received much but gave little neighboring behavior compared to those who gave much but received little neighboring behavior. This supports Hypothesis 2. To interpret the results holistically, coefficient estimates, standard errors, and covariances in the polynomial regression analysis were used. The overall response surface within the data range was plotted by adopting the method developed by Shanock et al. (59) (see Figure 2).

**Figure 3:**
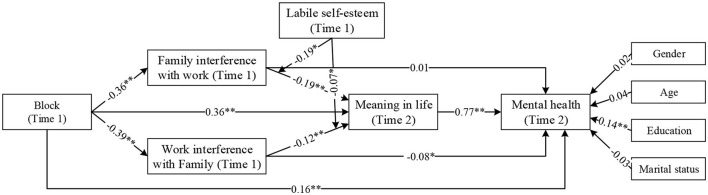
Results of block approach path analysis. **p < 0.01, *p < 0.05.

To assess the underlying spillover process that links community resources to mental health, this study used a block variable approach as specified by Edward and Cable (57) to test hypotheses H3–H6. The results are shown in Figure 3. Receiving and giving neighboring behavior fit was negatively correlated with both family interference with work (β = −0.36, *p* < 0.01) and work interference with family (β = −0.39, *p* < 0.01). This supports Hypothesis 3. Family interference with work (β = −0.19, *p* < 0.01) and work interference with family (β = −0.12, *p* < 0.01) were negatively associated with meaning in life. This supports Hypothesis 4. Meaning in life was positively associated with mental health (β = 0.77, *p* < 0.01), which supports Hypothesis 5.

To test hypothesis 6, a bootstrapping test was adopted, the results of which are shown in Table 4. The serial mediating effect was significant for both the family interference with work path (Effect = 0.11, SE = 0.03, 95%CI = [0.06, 0.18]) and the work interference with family path (Effect = 0.08, SE = 0.03, 95%CI = [0.03, 0.14]).

**Table 4:** Results of bootstrapping analysis.

			**Confidence interval**	
	**Effect**	**SE**	**95%LL**	**95%UL**
**Mediation effect**				
Family interference with work path	0.11	0.03	0.06	0.18
Work interference with family path	0.08	0.03	0.03	0.14
**Moderated effect of labile self-esteem**				
Family interference with work to meaning in life				
Low labile self-esteem (M-SD)	−0.01	0.04	−0.08	0.06
High labile self-esteem (M+SD)	−0.28	0.04	−0.35	−0.21
Difference	−0.27	0.05	−0.37	−0.18
Work interference with family to meaning in life				
Low labile self-esteem (M-SD)	−0.04	0.04	−0.12	0.05
High labile self-esteem (M+SD)	−0.17	0.04	−0.25	−0.08
Difference	−0.13	0.06	−0.24	−0.02
**Moderated meditation model**				
Family interference with work path				
Low labile self-esteem (M-SD)	0.00	0.03	−0.05	0.06
High labile self-esteem (M+SD)	0.23	0.05	0.13	0.33
Difference	0.22	0.06	0.12	0.34
**Work interference with family path**				
Low labile self-esteem (M-SD)	0.03	0.03	−0.04	0.09
High labile self-esteem (M+SD)	0.13	0.04	0.06	0.21
Difference	0.10	0.05	0.01	0.20

**Figure 4:**
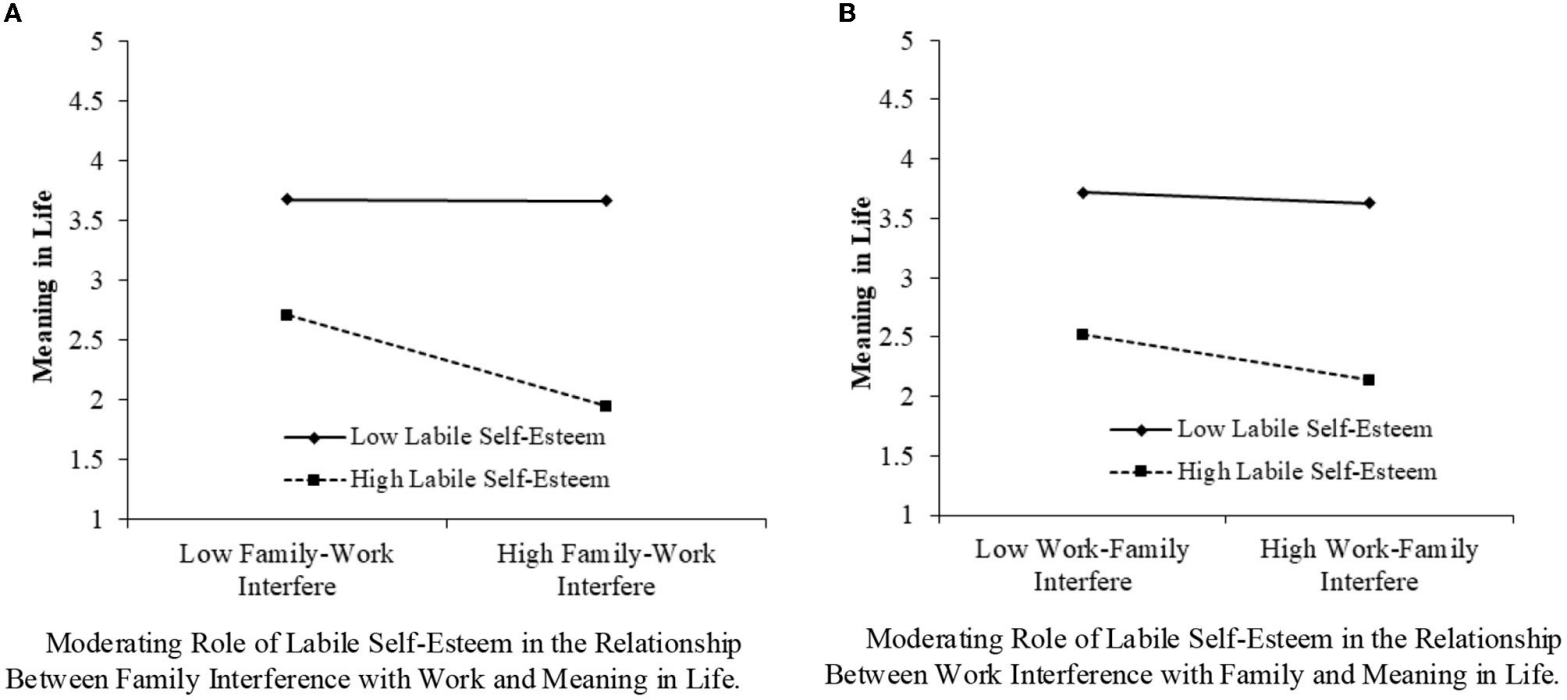
The moderating role of labile self-esteem.

To test Hypotheses 7 and 8, two interactive items were added to the path analysis. The interactive item of labile self-esteem with family interference with work was significant (β = −0.19, *p* < 0.01). A simple slope test was further conducted to assess the moderating effect. The results indicate that the negative influence of family interference with work on meaning in life emerged for working residents with high labile self-esteem (Effect = −0.28, SE = 0.04, 95%CI = [−0.35, −0.21]). The moderating effect of labile self-esteem in the relationship between family interference with work and meaning in life is shown in Figure 4.

The interactive item of labile self-esteem with work interference with family was significant (β = −0.07, *p* < 0.05). A simple slope test was further conducted to assess the moderating effect. The results indicate that a negative influence of work interference with family on meaning in life affected working residents with high labile self-esteem (Effect = −0.17, SE = 0.04, 95%CI = [−0.25, −0.08]). The moderating effect of labile self-esteem in the relationship between work interference with family and meaning in life is shown in Figure 4. This supports Hypothesis 7.

Furthermore, the moderated mediation model was examined through bootstrapping tests. The results shown in Table 4 indicate that the indirect effect of giving and receiving neighboring behavior fit on mental health through the family interference with work path affected working residents with high labile self-esteem (Effect = 0.23, SE = 0.05, 95%CI = [0.13, 0.33]). Also, the indirect effect of giving and receiving neighboring behavior fit on mental health through the work interference with family path affected working residents with high labile self-esteem (Effect = 0.13, SE = 0.04, 95%CI = [0.06, 0.21]).

## Discussion

Based on ecological systems theory, this paper explores how both congruence and incongruence of receiving and giving neighboring behavior impact mental health. By employing polynomial regression and response surface analysis, this study found that working residents have a higher level of mental health when receiving and giving behavior are highly congruent. When receiving and giving neighboring behavior are imbalanced, it is better for working residents to receive rather than give neighboring behavior in order to maintain their mental health. Furthermore, this paper explored the serial mediating roles of work-family interference and meaning in life in the relationship between receiving and giving neighboring behavior fit and mental health. However, one result should be further explained. Polynomial regression analysis indicated that when mental health was regressed on receiving and giving neighboring behavior simultaneously, the receiving of neighboring behavior was positively associated with mental health. This is in line with prior studies demonstrating the positive influences of community support. However, the association between giving neighboring behavior and mental health was negative, which is not consistent with the findings of Zhang et al. (5). Voydanoff (15, 16) regarded the giving of neighboring behavior as a demand. Although giving neighboring behavior may yield social functioning for working residents, it also potentially occupies their time and cognitive capabilities and undermines their recovery experiences. In particular, when the resources they received from their living communities, namely receiving neighboring behavior, were controlled, the negative aspect of giving neighboring behavior emerged.

This study offers three contributions to the literature. First, this study explores the relationship between receiving and giving neighboring behavior fit and mental health, thus extending our understanding of the antecedents of mental health in the community. Prior studies have explored how giving neighboring behavior impacts mental health. Zhang et al. (5) indicated that working residents can realize self-development by helping neighbors. Their research suggests that social functioning is developed in the process of helping neighbors, thereby conveying capabilities that can be used to reduce work-family conflict and facilitate thriving at work. In addition to the positive aspects of neighboring behavior, its positive association with ego depletion has also been reported (15, 60). A potential explanation for these inconsistent results may be the neglect of working residents' receiving neighboring behavior. Giving neighboring behavior is interpersonal and reciprocal (61). It acts as an effective tool for cultivating social capital. Driven by norms of reciprocity, neighbors are motivated to provide support for focal working residents. Moreover, Zu et al. indicated the benefits of receiving neighboring behavior in relieving work-family conflicts and facilitating mental health (22). Therefore, this paper explored how receiving and giving neighboring behavior fit impacts mental health. Our results explain the paradoxical results from prior studies on giving neighboring behavior and provide a research paradigm for this stream of research.

Second, this paper uncovers the spillover process by which receiving and giving neighboring behavior fit impacts mental health by examining the serial mediating roles of work-family interference and meaning in life. Ecological systems theory suggests that resources are mobile and can be transferred to other domains. For example, Zhang et al. explored how community resources are transferred into the family and work domains (5). Following this logic, this study focused on two paths: the family interference with work path and the work interference with family path. These two paths reflect how community resources (i.e., neighboring behavior) are transferred into the family and work domains, respectively (1, 62). The results indicate that resources aggregated by neighboring behavior can be used to fulfill the demands of working residents' work and family roles, thereby enhancing both their meaning in life and mental health. Based on prior studies, this research replicated the spillover effect suggested by Griggs et al. (1) and Zhang et al. (4) that links receiving neighboring behavior and mental health. Moreover, this study extended this line of research by introducing congruence and incongruence in receiving and giving neighboring behavior as indicators of community resources.

Third, this study revealed the boundary condition under which community resources can be adopted to resolve work-family interference and enhance mental health. Most studies developed within the framework of ecological systems theory have regarded personality traits as moderators. In line with prior studies, this study introduced labile self-esteem into the transformation process of community resources. Labile self-esteem reflects the instability of self-esteem, which is commonly regarded as an indicator of depressive symptoms (63). As mentioned previously, individuals with labile self-esteem are more likely to be impacted by stressors (41). This paper verifies that working residents with high labile self-esteem are more sensitive to stress. Bakker and Demerouti suggested that job resources help employees who are experiencing high levels of job demands (64). The present study found that receiving and giving neighboring behavior fit can be used as a resource to fulfill both work and family demands in working residents with high labile self-esteem. By doing so, this study extended the study of Bakker and Demerouti (64) by determining when community resources are beneficial for working residents to maintain their mental health.

In summary, this study provides a novel perspective for discussing the association between neighboring behavior and mental health within the framework of ecological systems theory. Prior studies have mainly explored the influences of receiving and giving neighboring behavior on mental health separately. By instead examining receiving and giving neighboring behavior fit, this study specified the conditions under which neighboring behavior provides or consumes psychological resources. Furthermore, this study revealed the family (family interference with work) and work (work interferences family) paths through which community resources cultivated by neighboring behavior can be transformed to nurture mental health. Moreover, this study demonstrated the key role of labile self-esteem in shaping this spillover process. In doing so, this research contributes to ecological systems theory by applying it to explain how and when neighboring behavior is beneficial for mental health.

Furthermore, the results of this research have several practical implications. This study indicated that when both the receiving and giving of neighboring behavior are at a high rather than a low level, working residents have higher levels of mental health. When receiving and giving neighboring behavior are imbalanced, working residents only have higher levels of mental health in the condition of receiving high levels of neighboring behavior while giving low levels of neighboring behavior. These results suggest that reciprocal interactions between neighbors in the community are beneficial. For managers of communities, community activating measures should be hosted by professional social workers to provide a platform for working residents to interact with each other and build social networks. To avoid work-family interference, social workers should also provide social support to working residents, thereby increasing their meaning in life and mental health. Moreover, these results indicate that the influences of (in)congruence in receiving and giving neighboring behavior on mental health are contingent upon labile self-esteem. To undermine the buffering role of labile self-esteem, managers may present lectures concerning self-compassion and mindfulness to help working residents maintain stable self-esteem (21, 65).

### Limitations and Future Research

The current study has several limitations that point to the following directions for future research. First, this study could not establish firm causal relationships. Receiving and giving neighboring behavior were not manipulated. Therefore, the causal impact of receiving and giving neighboring behavior fit on mental health could not be inferred. Future research may adopt an experimental design and cross-lagged panel design to overcome this shortcoming.

Second, this study could not rule out common method variance [CMV; (66)]. Although a two-wave questionnaire survey design was adopted, CMV still led to potential bias in the results. The employed questionnaires were self-reported and more than one questionnaire was completed at the same time point (e.g., meaning in life and mental health). This may have introduced CMV bias into the results. Future research may adopt a multi-source survey design or objective data to avoid CMV. For example, giving neighboring behavior can be assessed by neighbors.

Third, this study was conducted within Chinese communities, which limits external validity. Collectivism culture is widespread in China (67), resulting in more widespread interactions between neighbors, especially among older working residents. Future research may replicate this study in different age groups and explore differences between Chinese and Western cultures.

## Data Availability Statement

The raw data supporting the conclusions of this article will be made available by the authors, without undue reservation.

## Ethics Statement

The studies involving human participants were reviewed and approved by Dalian University of Technology. The patients/participants provided their written informed consent to participate in this study.

## Author Contributions

JX contributed to supervision and funding acquisstion. ZZ performed the statistical analysis and wrote the first draft of the manuscript. YF contributed to the supervision of the study. JZ contributed to the design and data curation of the study. All authors contributed to manuscript revision, read, and approved the submitted version.

## Funding

This study was supported by Academic Innovation Program of University of Chinese Academy of Social Sciences (2021-KYLX02-02), Fundamental Research Funds for the Central Universities (DUT21RC(3)089), National Natural Science Foundation of China (Grant no. 71701083), and Basic Research Planning Project of Yunnan Province (Grant no. 2019FB084).

## Conflict of Interest

The authors declare that the research was conducted in the absence of any commercial or financial relationships that could be construed as a potential conflict of interest.

## Publisher's Note

All claims expressed in this article are solely those of the authors and do not necessarily represent those of their affiliated organizations, or those of the publisher, the editors and the reviewers. Any product that may be evaluated in this article, or claim that may be made by its manufacturer, is not guaranteed or endorsed by the publisher.

## References

[1] Griggs TL Casper WJ Eby LT. Work, family and community support as predictors of work–family conflict: a study of low-income workers. J Vocat Behav. (2013) 82:59–68. 10.1016/j.jvb.2012.11.006

[2] Silva MJ de Huttly SR Harpham T Kenward MG. Social capital and mental health: a comparative analysis of four low income countries. Soc Sci Med. (2007) 64:5–20. 10.1016/j.socscimed.2006.08.044 17045716

[3] Perkins DD Florin P Rich RC Wandersman A Chavis DM. Participation and the social and physical environment of residential blocks: crime and community context. Am J Comm Psychol. (1990) 18:83–115. 10.1007/BF00922690

[4] Zhang Z Zhang L Zu X Liu T Zheng J. From neighboring behavior to mental health in the community: the role of gender and work-family conflict. Int J Environ Res Public Health. (2019) 16:22101. 10.3390/ijerph16122101 31200572 PMC6617099

[5] Zhang Z Li P Zhang L Zheng J Xue Z. Helping neighbors and enhancing yourself: a spillover effect of helping neighbors on work-family conflict and thriving at work. Current Psychology. (2020) 4:864. 10.1007/s12144-020-00864-4

[6] Zu X Zhang Z Wu Y Zheng J. The spillover effects of supportive neighboring behavior on mental health and career satisfaction: a longitudinal research on Chinese low-income employees. Psychol Res Behav Manag. (2020) 13:507–15. 10.2147/PRBM.S239435 32607020 PMC7304670

[7] Galanti T Guidetti G Mazzei E Zappalà S Toscano F. Work from home during the COVID-19 outbreak: the impact on employees' remote work productivity, engagement, and stress. J Occup Environ Med. (2021) 63:e426–32. 10.1097/JOM.0000000000002236 33883531 PMC8247534

[8] Sewell G Taskin L. Out of sight, out of mind in a new world of work? Autonomy, control, and spatiotemporal scaling in telework. Org Stu. (2015) 36:1507–29. 10.1177/0170840615593587

[9] Hunter EM Clark MA Carlson DS. Violating work-family boundaries: reactions to interruptions at work and home. J Manag. (2019) 45:1284–308. 10.1177/0149206317702221

[10] Crain TL Stevens SC. Family-supportive supervisor behaviors: a review and recommendations for research and practice. J Org Behav. (2018) 39:869–88. 10.1002/job.2320

[11] Vaziri H Casper WJ Wayne JH Matthews RA. Changes to the work-family interface during the COVID-19 pandemic: examining predictors and implications using latent transition analysis. J App Psychol. (2020) 105:1073–87. 10.1037/apl0000819 32866024

[12] Leung L Zhang R. Mapping ICT use at home and telecommuting practices: a perspective from work/family border theory. Telemat Inform. (2017) 34:385–96. 10.1016/j.tele.2016.06.001

[13] Hampton K Wellman B. Neighboring in Netville: how the internet supports community and social capital in a wired suburb. City Community. (2003) 2:277–311. 10.1046/j.1535-6841.2003.00057.x

[14] Zhang G Chen S Fan Y Dong Y. Influence of leaders' loneliness on voice-taking: the role of social self-efficacy and performance pressure. Int J Mental Health Prom. (2019) 21:13–29. 10.32604/IJMHP.2019.010730

[15] Gabriel AS Koopman J Rosen CC Johnson RE. Helping others or helping oneself? An episodic examination of the behavioral consequences of helping at work. Personnel Psychology. (2018) 71:85–107. 10.1111/peps.12229

[16] Voydanoff P. The effects of community demands, resources, and strategies on the nature and consequences of the work-family interface: an agenda for future research. Fam Relat. (2005) 54:583–95. 10.1111/j.1741-3729.2005.00343.x

[17] Voydanoff P. The differential salience of family and community demands and resources for family-to-work conflict and facilitation. J Fam Econ Issues. (2005) 26:395–417. 10.1007/s10834-005-5904-7

[18] Chamratrithirong A Lucktong A Jampaklay A Ford K. An analysis of the resilience process: the stimulus of mental strength and the role of community and family support amidst the civil violence in Thailand. Curr Psychol. (2020). 10.1007/s12144-020-01002-w

[19] Mostafa AM. The moderating role of self-sacrificing disposition and work meaningfulness on the relationship between work-family conflict and emotional exhaustion. J Happiness Stud. (2022) 23:1579–97. 10.1007/s10902-021-00463-5

[20] Shiah YJ Chang F Chiang SK Lin IM Tam WCC. Religion and health: Anxiety, religiosity, meaning of life and mental health. J Relig Health. (2015) 54:35–45. 10.1007/s10943-013-9781-3 24132457

[21] Veneziani CA Fuochi G Voci A. Self-compassion as a healthy attitude toward the self: factorial and construct validity in an Italian sample. Pers Individ Dif. (2017) 119:60–8. 10.1016/j.paid.2017.06.028

[22] Zu X Wu Y Song Y Zhang Z. The effect of received neighboring behavior on general health: The mediating role of work-family conflict. Asia Pac J Public Health. (2020) 32:250–7. 10.1177/1010539520928187 32551984

[23] Koopman J Lanaj K Scott BA. Integrating the bright and dark sides of OCB: a daily investigation of the benefits and costs of helping others. Aca Manag J. (2016) 59:414–35. 10.5465/amj.2014.0262

[24] Zhang Z Xiao H Zhang L Zheng J. Linking cyberbullying to job strain: roles of ego depletion and self-efficacy. J Aggre, Maltreat Trauma. (2021) 8:1–18. 10.1080/10926771.2021.1933288

[25] Carlson DS Kacmar KM Williams LJ. Construction and initial validation of a multidimensional measure of work–family conflict. J Vocat Behav. (2000) 56:249–76. 10.1006/jvbe.1999.1713

[26] Bennett MM Beehr TA Ivanitskaya LV. Work-family conflict: differences across generations and life cycles. J Manag Psychol. (2017) 32:314–32. 10.1108/JMP-06-2016-0192

[27] Johnson RC Eatough EM Chang C-H Hammer LB Truxilllo D. Home is where the mind is: family interference with work and safety performance in two high risk industries. J Vocat Behav. (2019) 110:117–30. 10.1016/j.jvb.2018.10.012

[28] Baskar A Mohansundaram A. A study on factors influencing the work interference with family and family interference with the work of selected cement industry workers. Int J Enginee Manag Res. (2018) 8:22. 10.31033/ijemr.8.4.22

[29] Las Heras M Rofcanin Y Matthijs Bal P Stollberger J. How do flexibility i-deals relate to work performance? Exploring the roles of family performance and organizational context. J Org Behav. (2017) 38:1280–94. 10.1002/job.2203

[30] Steger MF Frazier P Oishi S Kaler M. The meaning in life questionnaire: assessing the presence of and search for meaning in life. J Couns Psychol. (2006) 53:80–93. 10.1037/0022-0167.53.1.80

[31] Carballo-Penela A Varela J Bande B. The direct and indirect effects of self-efficacy on salespeople's emotional exhaustion and work-family conflict: a study using the job demands-resources model. Canadian J Administ Sci / Revue Canadienne des Sciences de l'Administration. (2019) 36:363–76. 10.1002/cjas.1503

[32] Smith TD Hughes K DeJoy DM Dyal MA. Assessment of relationships between work stress, work-family conflict, burnout and firefighter safety behavior outcomes. Saf Sci. (2018) 103:287–92. 10.1016/j.ssci.2017.12.005

[33] Nauman S Zheng C Naseer S. Job insecurity and work–family conflict: a moderated mediation model of perceived organizational justice, emotional exhaustion and work withdrawal. Int J Conflict Manag. (2020) 31:729–51. 10.1108/IJCMA-09-2019-0159

[34] Hicks JA King LA. Meaning in life and seeing the big picture: positive affect and global focus. Cogn Emot. (2007) 21:1577–84. 10.1080/02699930701347304

[35] Li Y. Building well-being among university teachers: the roles of psychological capital and meaning in life. Eu J Work Org Psychol. (2018) 27:594–602. 10.1080/1359432X.2018.1496909

[36] Pu J Hou H Ma R Sang J. The effect of psychological capital between work-family conflict and job burnout in Chinese university teachers: testing for mediation and moderation. J Health Psychol. (2017) 22:1799–807. 10.1177/1359105316636950 27030731

[37] Li PF Wong YJ Chao RC-L. Happiness and meaning in life: Unique, differential, and indirect associations with mental health. Couns Psychol Q. (2019) 32:396–414. 10.1080/09515070.2019.1604493

[38] Hamby S Grych J Banyard V. Resilience portfolios and poly-strengths: identifying protective factors associated with thriving after adversity. Psychol Violence. (2018) 8:172–83. 10.1037/vio0000135

[39] Kinnunen U Vermulst A Gerris J Mäkikangas A. Work–family conflict and its relations to well-being: the role of personality as a moderating factor. Pers Individ Dif. (2003) 35:1669–83. 10.1016/S0191-8869(02)00389-6

[40] Webster GD Smith CV Brunell AB Paddock EL Nezlek JB. Can Rosenberg's. (1965). Stability of Self Scale capture within-person self-esteem variability? Meta-analytic validity and test–retest reliability. J Res Personal. (2017) 69:156–69. 10.1016/j.jrp.2016.06.005

[41] Roberts JE Monroe SM. Vulnerable self-esteem and depressive symptoms: prospective findings comparing three alternative conceptualizations. J Pers Soc Psychol. (1992) 62:804–12. 10.1037/0022-3514.62.5.804 1593420

[42] Roberts JE Kassel JD. Labile self-esteem, life stress, and depressive symptoms: prospective data testing a model of vulnerability. Cognit Ther Res. (1997) 21:569–89. 10.1023/A:1021861503072

[43] Lee S Kim SL Park EK Yun S. Social support, work-family conflict, and emotional exhaustion in South Korea. Psychol Rep. (2013) 113:619–34. 10.2466/21.14.PR0.113x23z3 24597453

[44] King LA Hicks JA Krull JL Del Gaiso AK. Positive affect and the experience of meaning in life. J Pers Soc Psychol. (2006) 90:179–96. 10.1037/0022-3514.90.1.179 16448317

[45] Brislin RW. Cross-Cultural Research Methods. In: Environment and Culture. Boston, MA: Springer (1980). p. 47–82.

[46] Gao F Luo N Thumboo J Fones C Li SC Cheung YB. Does the 12-item General Health Questionnaire contain multiple factors and do we need them? Health Qual Life Outcomes. (2004) 2:63. 10.1186/1477-7525-2-63 15538951 PMC534792

[47] Grzywacz JG Marks NF. Reconceptualizing the work–family interface: an ecological perspective on the correlates of positive and negative spillover between work and family. J Occup Health Psychol. (2000) 5:111–26. 10.1037/1076-8998.5.1.111 10658890

[48] Crego A Yela JR Gómez-Martínez MÁ Karim AA. The contribution of meaningfulness and mindfulness to psychological well-being and mental health: a structural equation model. J Happiness Stud. (2020) 21:2827–50. 10.1007/s10902-019-00201-y

[49] Dykman BM. Integrating cognitive and motivational factors in depression: Initial tests of a goal-orientation approach. J Pers Soc Psychol. (1998) 74:139–58. 10.1037/0022-3514.74.1.139 9457779

[50] Rosander M Salin D Viita L Blomberg S. Gender matters: workplace bullying, gender, and mental health. Front Psychol. (2020) 11:560178. 10.3389/fpsyg.2020.560178 33123044 PMC7573240

[51] Bruine de Bruin W Isaacowitz D. Age differences in COVID-19 risk perceptions and mental health: Evidence from a national U.S. survey conducted in March 2020. J Gerontol: Series B. (2020) 76:e24-e29. 10.1093/geronb/gbaa074 32470120 PMC7542924

[52] Pokhilenko I Janssen LMM Evers SMAA Drost RMWA Simon J König H-H. Exploring the identification, validation, and categorization of costs and benefits of education in mental health: the PECUNIA project. Int J Technol Assess Health Care. (2020) 36:325–31. 10.1017/S0266462320000203 32715991

[53] Edwards JR. The study of congruence in organizational behavior research: critique and a proposed alternative. Organ Behav Hum Decis Process. (1994) 58:51–100. 10.1006/obhd.1994.1029

[54] Edwards JR Parry ME. On the use of polynomial regression equations as an alternative to difference scores in organizational research. Aca Manag J. (1993) 36:1577–613. 10.5465/256822

[55] Dawson JF. Moderation in management research: what, why, when, and how. J Bus Psychol. (2014) 29:1–19. 10.1007/s10869-013-9308-7

[56] Harris MM Anseel F Lievens F. Keeping up with the Joneses: a field study of the relationships among upward, lateral, and downward comparisons and pay level satisfaction. J Appl Psychol. (2008) 93:665–73. 10.1037/0021-9010.93.3.665 18457494

[57] Edwards JR Cable DM. The value of value congruence. J Appl Psychol. (2009) 94:654–77. 10.1037/a0014891 19450005

[58] Qin X Liu X Brown JA Zheng X Owens BP. Humility harmonized? Exploring whether and how leader and employee humility (in)congruence influences employee citizenship and deviance behaviors. J Busi Ethics. (2021) 170:147–65. 10.1007/s10551-019-04250-4

[59] Shanock LR Baran BE Gentry WA Pattison SC Heggestad ED. Polynomial regression with response surface analysis: a powerful approach for examining moderation and overcoming limitations of difference scores. J Bus Psychol. (2010) 25:543–54. 10.1007/s10869-010-9183-4

[60] Koopman J Rosen CC Gabriel AS Puranik H Johnson RE Ferris DL. Why and for whom does the pressure to help hurt others? Affective and cognitive mechanisms linking helping pressure to workplace deviance. Personnel Psychology. (2020) 73:333–62. 10.1111/peps.12354

[61] Deckop JR Cirka CC Andersson LM. Doing unto others: the reciprocity of helping behavior in organizations. J Busi Ethics. (2003) 47:101–13. 10.1023/A:1026060419167

[62] Frone MR Russell M Barnes GM. Work–family conflict, gender, and health-related outcomes: a study of employed parents in two community samples. J Occup Health Psychol. (1996) 1:57–69. 10.1037/1076-8998.1.1.57 9547034

[63] Hall HK Hill AP Appleton PR Kozub SA. The mediating influence of unconditional self-acceptance and labile self-esteem on the relationship between multidimensional perfectionism and exercise dependence. Psychol Sport Exerc. (2009) 10:35–44. 10.1016/j.psychsport.2008.05.003

[64] Bakker AB Demerouti E. The job demands-resources model: state of the art. J Manag Psychol. (2007) 22:309–28. 10.1108/0268394071073311531861812

[65] Bajaj B Gupta R Sengupta S. Emotional stability and self-esteem as mediators between mindfulness and happiness. J Happiness Stud. (2019) 20:2211–26. 10.1007/s10902-018-0046-4

[66] Podsakoff PM MacKenzie SB Lee JY Podsakoff NP. Common method biases in behavioral research: a critical review of the literature and recommended remedies. J Appl Psychol. (2003) 88:879–903. 10.1037/0021-9010.88.5.879 14516251

[67] Chen S Fan Y Zhang G Zhang Y. Collectivism-oriented human resource management on team creativity: effects of interpersonal harmony and human resource management strength. Int J Human Res Manag. (2021) 32:3805–32. 10.1080/09585192.2019.1640765

